# Enhancing prediction accuracy for enzyme activity engineering through sequence co-evolution and epistatic relationship modeling

**DOI:** 10.1093/nar/gkag879

**Published:** 2026-09-08

**Authors:** Da Neub Kim, Hyeongseop Kim, Donghyo Kim, Gyoo Yeol Jung, Sanguk Kim, Myung Hyun Noh

**Affiliations:** School of Interdisciplinary Bioscience and Bioengineering, Pohang University of Science and Technology, Pohang, Gyeongbuk, 37673, Korea; Center for Bio-based Chemistry, Korea Research Institute of Chemical Technology, Ulsan, 44429, Korea; School of Interdisciplinary Bioscience and Bioengineering, Pohang University of Science and Technology, Pohang, Gyeongbuk, 37673, Korea; Department of Chemical Engineering, Pohang University of Science and Technology, Pohang, Gyeongbuk, 37673, Korea; School of Interdisciplinary Bioscience and Bioengineering, Pohang University of Science and Technology, Pohang, Gyeongbuk, 37673, Korea; Department of Chemical Engineering, Pohang University of Science and Technology, Pohang, Gyeongbuk, 37673, Korea; School of Interdisciplinary Bioscience and Bioengineering, Pohang University of Science and Technology, Pohang, Gyeongbuk, 37673, Korea; Department of Life Sciences, Pohang University of Science and Technology, Pohang, Gyeongbuk, 37673, Korea; Center for Bio-based Chemistry, Korea Research Institute of Chemical Technology, Ulsan, 44429, Korea

## Abstract

Conventional enzyme engineering strategies, such as directed evolution with structure-based analysis, are limited by laborious workflows and by the need to screen extensive enzyme libraries. Computational models based on multiple sequence alignment, such as SCANEER, have streamlined these engineering strategies, enabling the successful prediction of single-site mutations that enhance enzyme activity. However, when combining predicted mutations to improve enzyme performance or expand mutational space, such approaches often fail due to epistatic interactions among amino acid residues, which can lead to complex, non-additive effects. Here, we present epiSCANEER, an extended computational framework that incorporates co-evolutionary dependencies reflecting residue-level epistatic effects to identify mutation combinations with a higher likelihood of enhancing enzyme activity. By evaluating amino acid pairs at co-evolved positions across homologous sequences, epiSCANEER prioritizes mutation combinations with high evolutionary compatibility, significantly narrowing the combinatorial search space to variants more likely to exhibit activity-enhancing effects. Experimental validation demonstrated a 76% success rate for epiSCANEER prediction, compared to 30% and 38% for single-site predictions and combinatorial approaches of single-site mutants, respectively. This novel method obviates the need to construct single-mutation libraries, significantly reducing labor and costs while improving success rates. epiSCANEER has been developed as a web-server that enables researchers to access tools for rational enzyme optimization.

## Graphical Abstract

**Graphical Abstract:**
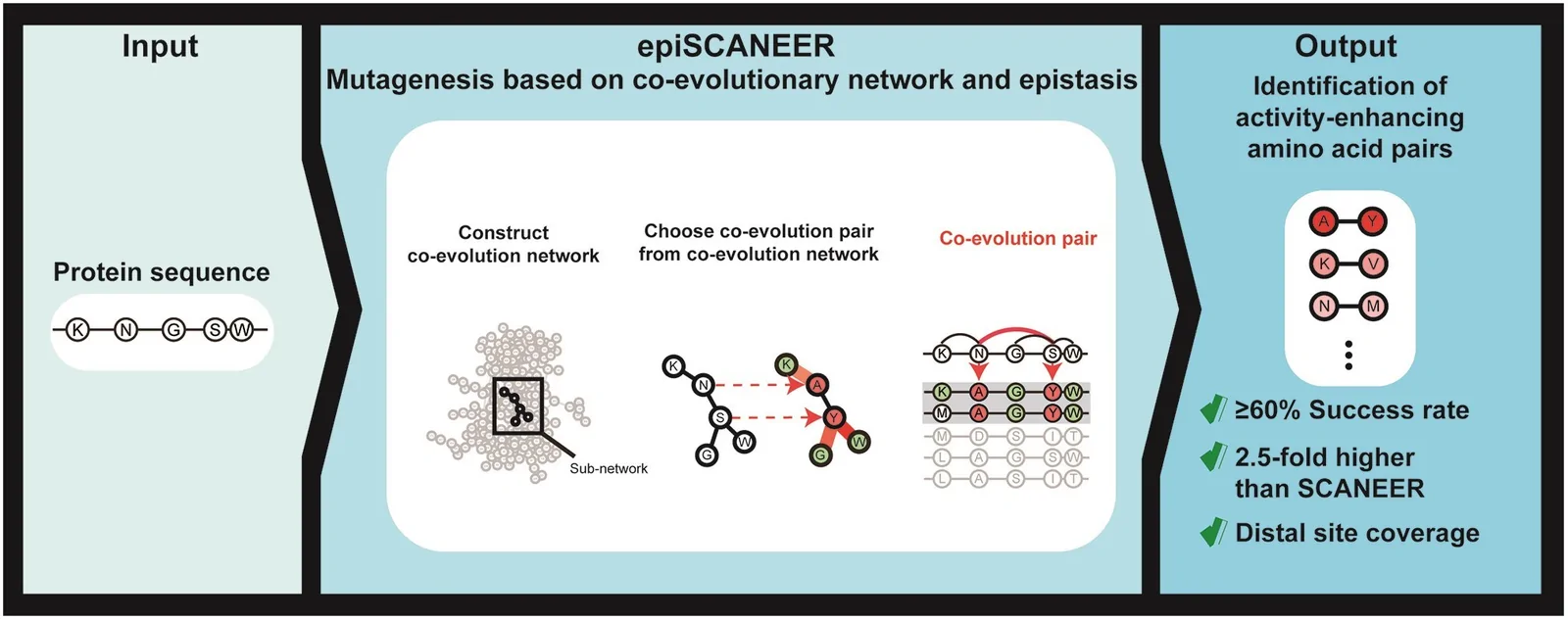

## Introduction

Enzyme engineering in synthetic biology possesses significant potential across diverse biosynthetic pathways, emerging as a promising field with substantial industrial applications [1–4]. Since native enzymes are often unsuitable for direct industrial application, enzyme engineering is necessary for improving their activity and properties [5, 6]. Conventional strategies have been achieved through directed evolution via random mutagenesis or structure-based rational approaches [7]. However, they entail limitations, including the intricate construction of large variant pools and reliance on biosensors for screening [8–12]. To overcome these constraints, computational approaches that predict alterations in enzyme activity prior to experimental validation have been actively studied in recent years [13, 14].

Among the prominent approaches are the evolution-based models utilizing multiple sequence alignment (MSA) [15–17]. These models are effective in identifying amino acid residues critical for enzyme activity, since native proteins have optimized their functions through selectively accumulating mutations to adapt to species-specific environments [18, 19]. Consequently, analysis of homologous protein sequences facilitates the prediction of functional residues that alter enzyme activity. A representative model, SCANEER, predicts single-site mutations affecting enzyme activity based on the hypothesis that amino acids contributing to beneficial mutations are encoded in the evolutionary history of protein sequences, while deleterious ones have been eliminated through natural selection [15].

Recent studies have focused on constructing combinatorial libraries from single-site mutations to obtain superior enzymes [20]. Effective single-site mutations often accompany reduced thermostability or decreased expression levels, while combinatorial mutations can mitigate the stability-function trade-off by incorporating compensatory mutations that confer structural stabilization alongside functional modification [21–23]. Combinatorial approaches may seem to yield the optimal enzyme by accumulating the effects of beneficial residues; however, they usually do not lead to improvements in activity. This occurs due to epistatic effects, where the functional impact of a mutation at one residue is non-additively modulated by mutations at other residues [24]. Consequently, certain combinations of mutations predicted to be beneficial at the single-site level may paradoxically reduce activity when epistasis is not accounted for, while potentially synergistic combinations of mutations that appear adverse at the single-site level may be overlooked. This constraint can trap engineering efforts in local maxima within the explorable sequence space, thereby negating the substantial cost and labor required to construct mutation libraries [25, 26]. Recently, approaches targeting active site substrate binding have addressed epistasis to some extent [27, 28]. However, enzyme activity is also substantially influenced by long-range interactions, such as allosteric regulation and conformational changes [29]. Therefore, a novel approach encompassing the global enzyme structure is necessary to provide structural flexibility for enzyme design.

In this study, we developed epiSCANEER, a model that prioritizes evolutionarily compatible double-site mutations, which experimentally exhibit a high likelihood of increasing enzyme activity, by leveraging co-evolutionary dependencies that reflect residue-level epistasis effects within the co-evolutionary networks. We hypothesized that incorporating residue–residue interaction information embedded in co-evolutionary networks would enable the prediction of residue combinations with high coevolutionary compatibility, thereby prioritizing candidates that are more likely to manifest as synergistic mutations under experimental validation by explicitly accounting for how each individual mutation influences coevolutionary constraints on other residues. This approach extends beyond considering the effects of single mutations in the context of residue relationships, by further capturing the mutual influence between paired mutations. Amino acid mutation pairs formed at co-evolved positions across homologous sequences were experimentally evaluated for their predictive accuracy in two enzymes. Furthermore, this approach provides mutation combinations at distal sites far from the active site and enables direct exploration of multi-site mutations without constructing single-site libraries, thereby enhancing success rates and efficiency while substantially reducing labor and cost. While we focus on double-site mutations to ensure statistical reliability and experimental feasibility, this method effectively identifies evolutionarily compatible mutation pairs consistent with epistatic constraints that are missed by single-site predictions. The model has been developed as a user-friendly web server that provides researchers with easy access to advanced enzyme engineering tools.

## Materials and methods

### MSA generation

Homologous protein sequences corresponding to the query were retrieved from the UniRef90 database, which consists of non-redundant protein sequences clustered at 90% sequence identity, using PSI-BLAST [30]. The PSI-BLAST search was performed with a single iteration and an e-value threshold of 0.001. To ensure the quality of the resulting MSAs, only sequences whose lengths ranged from 0.7 to 1.3 times that of the query sequence were retained, and sequences with 90% or more identity to the query were excluded. MSAs were constructed using MUSCLE with the ClustalW output format and default parameter settings [31]. Because the proposed method is sensitive to the composition and quality of the MSA, users can manually curate or provide custom MSAs to obtain results tailored to their specific objectives.

### Construction of coevolutionary networks by SCANEER

To construct a coevolutionary network that represents coevolutionary relationships among enzyme residues, we adopted the methodology implemented in the SCANEER model. This approach quantifies the co-varying strength between pairs of distinct residue positions based on MSAs using the McLachlan-based substitution correlation method [32]. Residue pairs were then ranked according to their co-varying strengths, and the top-ranked pairs corresponding to twice the protein length were selected and defined as significantly co-evolving residue pairs. This procedure enables the effective extraction of statistically meaningful coevolutionary relationships [33–35]. In the resulting coevolutionary network, each node represents an individual residue within the enzyme, and an edge indicates a coevolutionary relationship between the connected residue pair.

### Computation of the Sequence Coevolution Index for mutation combination

The Sequence Coevolution Index (SCI) for double-site mutations was computed based on changes in mutual information within coevolutionary networks. Building upon the SCANEER hypothesis that coevolving amino acid pairs with functional significance exhibit evolutionarily constrained variability, we defined the entropy difference between wild-type and mutant residue pairs at positions *i* and *j* as follows:


\begin{equation*}\Delta {{S}_{i,\ j}}\left( {\alpha \rightarrow\gamma ,\ \beta \rightarrow\delta } \right) = \mathrm{ ln}\left( {\frac{{\mathrm{ MU{{T}}_{\mathrm{ num}}} + 1}}{{\mathrm{ W{{T}}_{\mathrm{ num}}}}}} \right).\end{equation*}

Here, *α* and *β* denote the wild-type amino acids at positions *i* and *j*, respectively, and *γ* and *δ* indicate the corresponding mutant residues. $\mathrm{ W{{T}}_{\mathrm{ num}}}$ and $\mathrm{ MUT}\_\mathrm{ num}$ represent the observed frequencies of (*α, β*) and (*γ, δ*) pairs in an MSA. To prevent undefined values arising from zero counts, pseudocount smoothing was applied.

To account for the propagation of mutational effects beyond the directly mutated sites, we incorporated the structural context of the coevolutionary network. For each mutated pair (*i, j*), a local coevolutionary subnetwork C was extracted, comprising all edges connected to nodes *i* and *j*. The cumulative entropy change across this subnetwork was then normalized by the total number of edges in the full coevolutionary network, yielding the final SCI score:


\begin{equation*}\mathrm{ SC}{{\mathrm{ I}}_{i,\ j\left( {\alpha \rightarrow\gamma ,\ \beta \rightarrow\delta } \right)}} = \frac{{\mathop \sum \nolimits_{i,\ j\in C} {\mathrm{\Delta }}{{S}_{i,\ j}}\left( {\alpha \rightarrow\gamma ,\ \beta \rightarrow\delta } \right)}}{{\left| {\mathrm{Edge}} \right|}}.\end{equation*}

This normalization ensures comparability of SCI scores across proteins of varying network sizes. Finally, SCI scores were min-max normalized to the [0, 1] range to facilitate candidate ranking across enzymes.

### Oligonucleotides and reagents

Oligonucleotides for cloning were synthesized by Macrogen (Seoul, Korea). DNA fragments were amplified with Q5 High-Fidelity DNA polymerase (New England Biolabs, Ipswich, MA, USA) and purified with the QIAquick PCR purification kit (Qiagen, Hilden, Germany). Plasmids were assembled using NEBuilder HiFi DNA Assembly Master Mix (NEB) and extracted with the Accuprep Nano-Plus Plasmid Mini Extraction Kit (BIONEER, Daejeon, Korea). Reagents for cell culture and enzyme assays were purchased from Sigma–Aldrich (St. Louis, MO, USA).

### Construction of strains and plasmids

All bacterial strains and plasmids are listed in Supplementary Table S1. *Escherichia coli* DH5α was utilized as a cloning host (BioFACT, Daejeon, Korea). The pACYC_B4, pQE80L-K, pACYC_galP_ldhA, and pCDF-3HPselector plasmids were obtained from previous studies [36, 37]. The pACYC_B4 plasmid encodes enzymes mediating the conversion of glycerol to 3-hydroxypropionaldehyde, and pQE80L-K encodes KGSADH (alpha-ketoglutaric semialdehyde dehydrogenase, UniProtKB: Q1JUP4), which converts 3-hydroxypropionaldehyde to 3-hydroxypropionic acid (3-HP; Supplementary Table S2). The pACYC_galP_ldhA encodes LdhA (lactate dehydrogenase A) from *Vibrio* sp. dhg, of which the amino acid sequence corresponds to UniParc entry UPI000E531CA4; a UniProtKB accession is not currently available for this sequence. The tetA gene (tetracycline resistance structural protein TetA, UniProtKB: Q79SH9) was amplified by polymerase chain reaction (PCR) from the pCDF-3HPselector plasmid and reassembled into a pACYC vector under the control of the J23113 promoter. A 6× His-tag was introduced into the C-terminus of the *ldhA* gene for His-tag purification, and the *galP* gene was deleted to prevent unnecessary expression in the pACYC_galP_ldhA plasmid. The plasmids for each mutant expression (pQE80L-K^mut^, pACYC_ldhA^mut^, and pACYC_J23113_tetAmut) were cloned using site-directed mutagenesis [38]. The *Escherichia coli* W strain was utilized for 3-HP production to construct the WBK strain, and the VΔ5 strain (*Vibrio* sp. dhg Δ*dns* Δ*ldhA* Δ*frdABCD* Δ*pflB* Δ*ack-pta*) was employed for lactate production to construct the VL strain [39]. Additionally, *E. coli* DH5α was utilized for tetracycline resistance selection, designated as the DT strain.

### Culture medium and conditions

3-HP production was evaluated in modified glycerol minimal medium containing 0.5 g/l MgSO_4_⋅7H_2_O, 2 g/l NH_4_Cl, 2 g/l NaCl, 1 g/l yeast extract, 100 mM potassium phosphate buffer (pH 7.0), and 100 mM glycerol as a carbon source [37]. To prepare a seed culture, each colony of the WBK strain was inoculated into 3 ml of medium in a 14-ml test tube. Overnight cell cultures were diluted into 3 ml of fresh medium in a 14-ml test tube to an optical density of 0.05 at 600 nm (OD_600_ = 0.05). 0.5 mM isopropyl-β-d-thiogalactopyranoside (IPTG) and 2 μM Coenzyme B_12_ were initially added for cell induction. The cultures were incubated for 36 h under continuous shaking (250 rpm) at 37°C.

Lactate production was performed in the same manner as the evaluation of 3-HP production unless otherwise specified. Sodium M9 minimal medium containing 14.6 g/l NaCl, 11.28 g/l M9 minimal salts, 2 mM MgSO_4_⋅7H_2_O, 0.1 mM CaCl_2_, 1 g/l yeast extract, and 10 g/l glucose as a carbon source was used for *Vibrio* sp. dhg cultivation. The overnight seed cultures were diluted into 10 ml of fresh medium in a 50-ml conical tube for semi-aerobic conditions, and the cultures were incubated for 12 h under continuous shaking (250 rpm) at 37°C.

TetA resistance was evaluated in Luria–Bertani (LB) medium supplemented with 5 µg/ml tetracycline. To prepare the seed culture, each colony of the DT strain was inoculated into 3 ml of LB medium in a 14-ml test tube. Overnight seed cultures were diluted into 600 µl of fresh medium in a 2-ml deep well plate (Biofil, Guangzhou, China), and the cultures were incubated for 36 h in an orbital shaker (LK Lab Korea, Gyonggi, Korea) (1000 rpm) at 37°C.

### Quantification of cell biomass and metabolites

A UV-2600 spectrophotometer (Shimadzu, Kyoto, Japan) was used to measure cell biomass at 600 nm. For the TetA resistance assay, optical density at 600 nm was measured using a SpectraMax 340PC384 Microplate Reader (Molecular Devices, CA, USA). Specific growth rates (*µ*) were calculated by linear regression of ln (OD_600_) versus time during the mid-exponential growth phase, identified using a sliding window approach across multiple time intervals. An HM-41X pH meter (TOADKK, Tokyo, Japan) was used to measure pH. Cellular metabolites were analyzed using an Agilent 1260 Infinity (Agilent Technologies, Santa Clara, CA, USA) with an Aminex HPX-87H column (Bio-Rad Laboratories, Hercules, CA). Liquid samples taken from cell cultures were centrifuged and filtered through a 0.2-μm syringe filter. The filtered samples were analyzed with a mobile phase of 5 mM H_2_SO_4_ at a flow rate of 0.6 ml/min. The refractive index signal was measured using an Agilent 1260 Infinity RID (Agilent).

### *In vitro* enzyme assay

The *E. coli* DH5α strain was utilized as the expression host for routine protein purification. Cells were cultured in the appropriate medium until OD_600_ reached 0.8 and lysed using a Qsonica sonicator (Newtown, CT, USA). The 6× His-tagged proteins within cell lysates were extracted using the MagListo His-tagged protein purification kit (Bioneer, Daejeon, Korea). The concentrations of purified enzymes were measured utilizing the Quick Start Bradford Protein Assay Kit (Bio-Rad Laboratories). Protein purity was validated by sodium dodecyl sulfate–polyacrylamide gel electrophoresis with the Mini-PROTEAN Tetra System (Bio-Rad Laboratories), as shown in Supplementary Fig. S1.

The enzyme assay of KGSADH was performed with slight modifications as described in a previous study [37]. The purified enzymes were added to reaction mixtures containing 100 mM phosphate buffer (pH 8), 1 mM ethylenediaminetetraacetic acid , 1 mM dithiothreitol (DTT), and 2 mM NAD^+^. Propionaldehyde was added as the substrate at various concentrations from 0.1 to 2 mM, monitoring the conversion rate of NAD^+^ to NADH with the Hidex Sense Microplate Reader 425-301 (Hidex, Turku, Finland) at 25°C [40]. The enzyme activity of KGSADH was calculated from the slope of the linear portion, where absorbance at 340 nm was monitored for 30 min. The enzyme kinetics of LdhA were determined in a reaction mixture containing 150 mM phosphate buffer (pH 6.5), 2.5 mM MgCl_2_, and 200 μM NADH [41]. Sodium pyruvate concentrations ranged from 10 to 200 μM, and NADH to NAD^+^ conversion was monitored using a Hidex Sense Microplate Reader. The enzyme activity of LdhA was calculated from the negative slope of the decrease in absorbance at 340 nm for 30 min.

### Definition of first- and second-shell residues and average distance calculation

Protein structural analyses were performed using atomic coordinates extracted from PDB files with the Biopython PDBParser [42]. Only standard amino acid residues were considered; heteroatoms and hydrogen atoms were excluded from all analyses. Target residues were specified by chain ID and residue sequence number. For each target residue, all heavy atoms were collected and used as reference atoms. A residue-level neighbor search was conducted using NeighborSearch implemented in Biopython, based on all atoms in the protein structure.

First-shell residues were defined as residues containing at least one atom located within 5.0 Å of any heavy atom belonging to the target residues. Second-shell residues were defined as residues containing at least one atom within 8.0 Å of any target heavy atom, excluding both the target residues themselves and residues already classified as first-shell residues. To quantify spatial separation between the target residues and each shell, an average distance metric was computed. For a given shell, all pairwise Euclidean distances were calculated between heavy atoms of the target residues and heavy atoms of residues belonging to the shell. The average of these atom–atom distances was used as the representative distance between the target residues and the corresponding shell. For each enzyme, we calculated the first- and second-shell residues relative to the active site, designating residues 253 and 287 as the active sites in KGSADH and residue 299 as the active site in LdhA.

## Results

### Prediction for double-site mutants to improve enzyme activity

**Figure 1.**
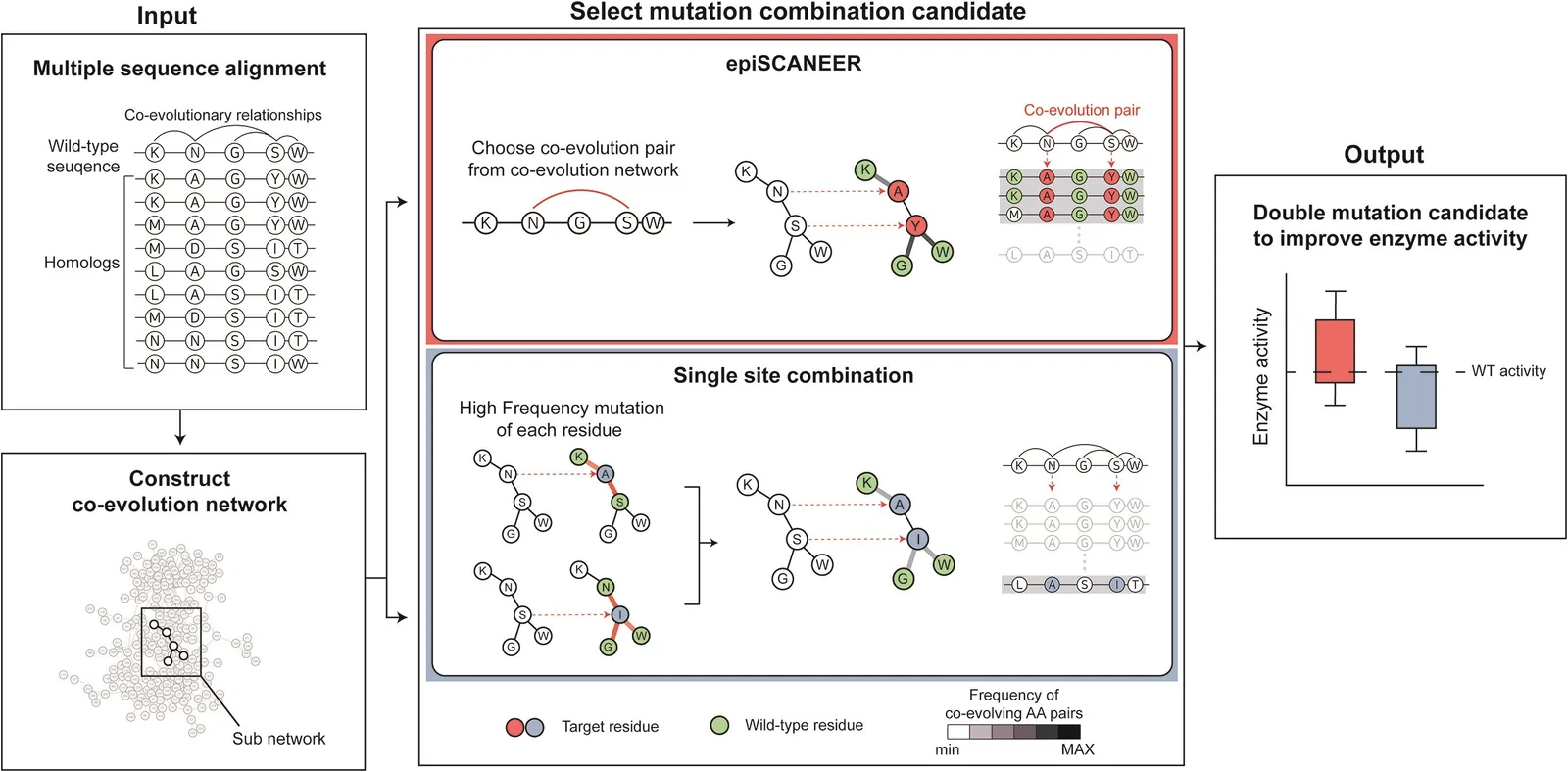
Framework of epiSCANEER for identifying activity-enhancing double mutations. Users input a query sequence or MSA, from which coevolutionary networks are constructed to extract residue pairs linked by coevolution. SCI scores, derived from MSA frequencies of amino acids at coevolving pairs upon simultaneous mutation, rank candidate combinations. By accounting for epistatic relationships, epiSCANEER prioritizes evolutionarily compatible double-mutation combinations, thereby enhancing the probability of identifying variants with superior activity over wild-type at a higher success rate than single-site combinations.

We constructed a predictive model that utilizes evolutionary information derived from MSA to prioritize double-mutation combinations with high evolutionary compatibility (Fig. 1). The MSA data encapsulate coevolutionary patterns between amino acid residues that have evolved in a mutually dependent manner. These patterns not only reflect spatially proximal interactions within the three-dimensional protein structure but also encode long-range relationships that contribute to protein dynamics and allosteric regulation. Such residue–residue interactions critically influence both the catalytic activity and structural stability of proteins. To quantitatively represent these coevolutionary relationships, we constructed a coevolution network and calculated the SCI, which incorporates pairwise dependencies inferred from co-evolutionary patterns. The SCI quantifies how evolutionarily favorable a pair of mutations is for maintaining the wild-type amino acids at other coevolving residues when both positions are mutated simultaneously. In other words, SCI assigns higher scores to mutation combinations that are less likely to induce loss-of-function effects. Consequently, this approach offers a distinct strategy for identifying evolutionarily compatible mutation combinations by minimizing distortions arising from nonlinear residue interactions consistent with epistasis that may occur when highly conserved residues are simply combined at the single-residue level.

### Validation of a double-site prediction model embedded with epistatic relationships

**Figure 2.**
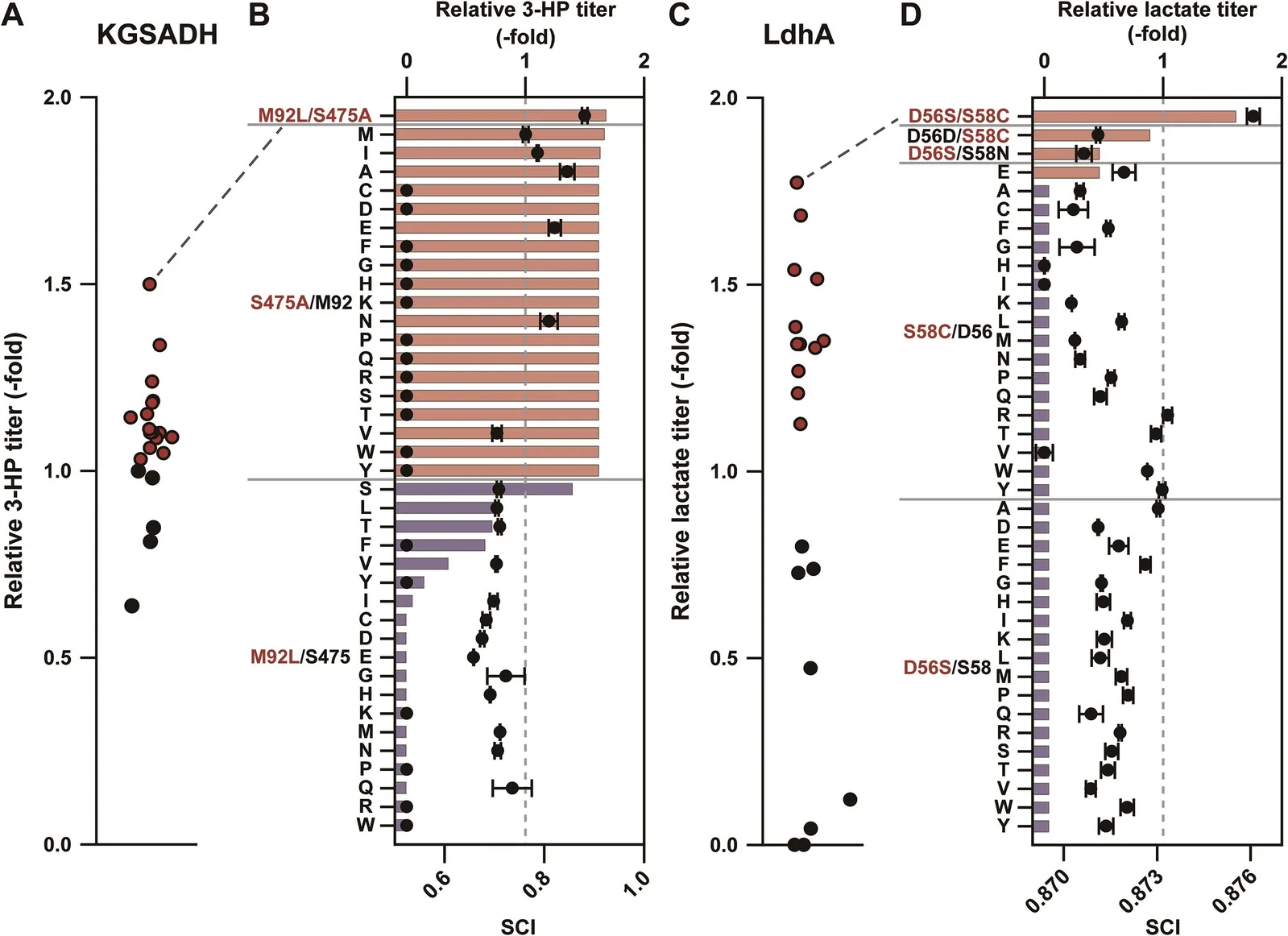
Experimental validation of double-site mutants predicted by epiSCANEER. (**A**) Enzyme activity validation of 21 KGSADH variants. Red dots indicate variants with a titer exceeding 1.0-fold relative to wild type. (**B**) Site-saturated mutagenesis at each position of M92L/S475A, with one residue fixed (indicated in red text), while the other position was substituted with all canonical amino acids. Variants are ordered in descending SCI score. Red and blue bars indicate SCI values above and below the threshold (0.86), respectively. The dashed vertical line indicates wild-type production titer (1.0-fold). (**C**) Enzyme activity validation of 20 LdhA variants. Same as panel (A). (**D**) Same as panel (B) for D56S/S58C, with SCI threshold of 0.87. All data represent biological replicates (*n* = 3) with error bars indicating standard deviation. All titer values are presented relative to wild type.

We developed epiSCANEER, a novel double-site prediction tool that extends the previous single-site model and evaluated its accuracy in predicting amino acid mutations that enhance the activity of chemical production enzymes (Fig. 2A–D). To prevent interference from irrelevant enzymes and achieve a direct correlation between enzyme activity and production titer *in vivo*, two industrial oxidoreductases, KGSADH and LdhA, were selected for 3-HP and lactate production, respectively (Fig. 2A and C, and Supplementary Fig. S2). A strain with deleted by-product pathways was utilized to enhance pyruvate availability for LdhA (Supplementary Table S1). Based on SCI values, strains expressing 21 and 20 enzyme mutants (for KGSADH and LdhA, respectively) were constructed. To minimize redundancy in mutation positions and ensure broad exploration of the mutational space across the predicted library, we used a top 5% SCI threshold for each enzyme (KGSADH:0.86; LdhA:0.87). Specifically, to determine an appropriate SCI threshold, all predicted double-site mutants were ranked by SCI score and stratified into 20 ranked groups (G1–G20). A representative variant from each group was experimentally validated, revealing that variants in the highest-ranked group (G20, corresponding to SCI ≥0.86 for KGSADH and ≥0.87 for LdhA) showed the highest titer improvements (Supplementary Fig. S2, Supplementary Tables S3 and S4). Interestingly, when comparing the predicted residues between the two models, the eight KGSADH double mutants with the highest SCI values comprised combinations of residues also identified by the previous single-site prediction model, whereas the remaining variants harbored newly predicted mutations not present in the single-site model (K35Q, M92L, V158I, S166G, V195A, V424A, and V478I in Supplementary Table S5). Furthermore, the current model did not predict superior mutations from single-site predictions, such as V18C, S109A, and R192Q, neither at the same residue nor at the same position, as these residues primarily manifest as isolated variations in homologous sequences rather than engaging in coevolutionary mutation pairs. When comparing the performance of strains with predicted mutants, 16 of 21 and 12 of 20 mutants showed titer increases with M92L/S475A (1.50-fold) and D56S/S58C (1.77-fold), respectively, achieving the greatest improvements for KGSADH and LdhA, respectively (Supplementary Tables S6 and S7).

Notably, the S475A mutation alone showed minimal activity improvement (1.00-fold), but when combined with M92L, it resulted in a 1.50-fold increase, demonstrating positive epistasis in which the combined effect significantly exceeds the individual contributions. Furthermore, the residues of M92L/S475A and D56S/S58C—which exhibited the highest titer increases—were substituted with other canonical amino acids at each position to assess whether epiSCANEER accurately predicted the optimal amino acid residues at each position. One residue was fixed to the predicted amino acid, while the other position underwent site-saturated mutagenesis to compare the performance of the proposed mutation candidates against alternative substitutions (Fig. 2B and D, and Supplementary Tables S8 and S9). The distribution of SCI values confirmed that this approach accounted for all 400 possible combinations of 20 residues at each position (Supplementary Fig. S3). In KGSADH, when M92L was fixed, and S475 was substituted with the other 19 amino acids, both SCI values and titers were predominantly low; conversely, when M92L was substituted, several variants showed titer increases, but most exhibited loss-of-function phenotypes. In LdhA, regardless of which position underwent site-saturated mutagenesis, all substitutions with lower SCI values resulted in decreased titers or loss of function. These results demonstrated that the residues at each position predicted by epiSCANEER were accurately identified.

### Combinatorial evaluation of single-site predicted mutations without epistatic modeling

**Figure 3.**
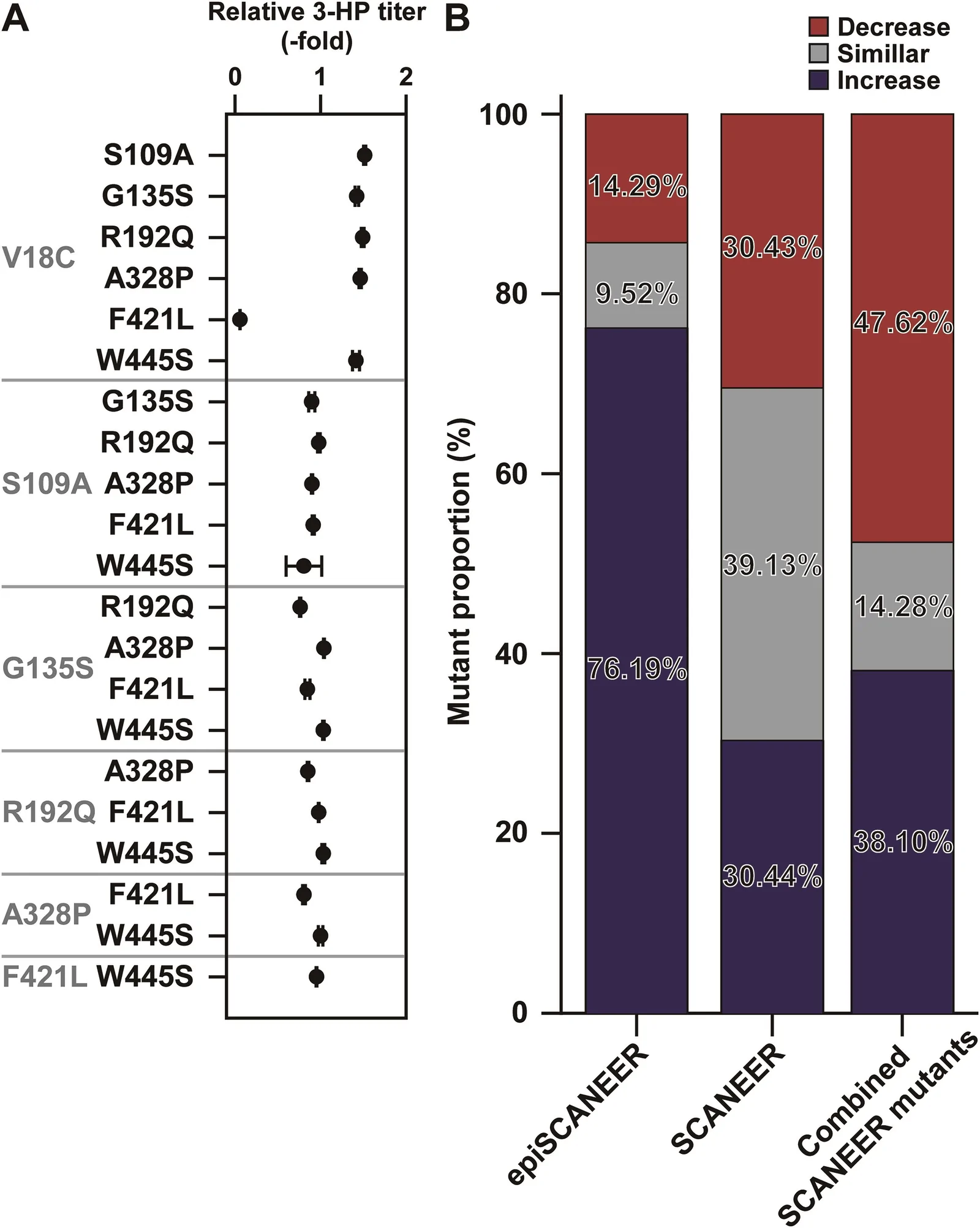
Evaluation of prediction accuracy derived from different models. (**A**) Random combinations of SCANEER-predicted single-site mutations and their enzyme activity validation. Mutations shared across variants are indicated in gray text. All data represent biological replicates (*n* = 3) with error bars. (**B**) Comparison of mutant proportions among epiSCANEER, SCANEER, and combined SCANEER mutants. Thresholds of decrease and increase are 0.97- and 1.03-fold, respectively.

To investigate whether explicitly accounting for epistatic relationships provides advantages over simply combining beneficial single-site mutations, we constructed combinatorial mutants using KGSADH mutations previously identified by SCANEER (Supplementary Table S10). Specifically, mutations V18C, S109A, G135S, R192Q, A328P, F421L, and W445S showed titer improvements ranging from 1.02- to 1.51-fold, and these were randomly combined to construct strains bearing 21 mutants (Supplementary Table S5 and Fig. 3A). The V18C mutation showed the most effective titer increase of 1.51-fold; five mutants containing V18C consistently showed titer increases above 1.41-fold in combination, although the V18C/F421L variant exhibited deleterious effects. Notably, the greatest increase, 1.52-fold, was observed when V18C was combined with the S109A mutation. In addition, combinations of other mutations also showed only a marginal impact, suggesting the need to account for epistatic relationships.

To thoroughly assess the prediction performance, enzyme activity distributions from the double-site prediction model, the single-site prediction model, and the combined single-site mutations were compared. This comparison highlights the improved accuracy of epiSCANEER, which incorporates epistasis (Fig. 3B). The epiSCANEER model achieved a success rate of 76.19% (defined as a 1.03-fold or greater titer increase), while SCANEER and the combined mutations achieved rates of 30.43% and 38.10%, respectively. Additionally, epiSCANEER produced a smaller proportion of deleterious variants (14.29%, defined by a titer reduction of 0.97-fold or less) compared to SCANEER (30.43%) and the combined mutations (47.62%). Furthermore, epiSCANEER exhibited a more concentrated distribution, with fewer variants causing loss-of-function. These findings underscore the importance of considering epistatic interactions when predicting mutations at multiple sites and demonstrate that double-site predictions can be highly accurate even across a broader mutational landscape. Overall, the epiSCANEER model has the potential to identify activity-enhancing variants with fewer experimental iterations, reducing labor costs and accelerating enzyme development.

### Structural localization of the proposed residues and their impact on enzyme kinetics

**Figure 4.**
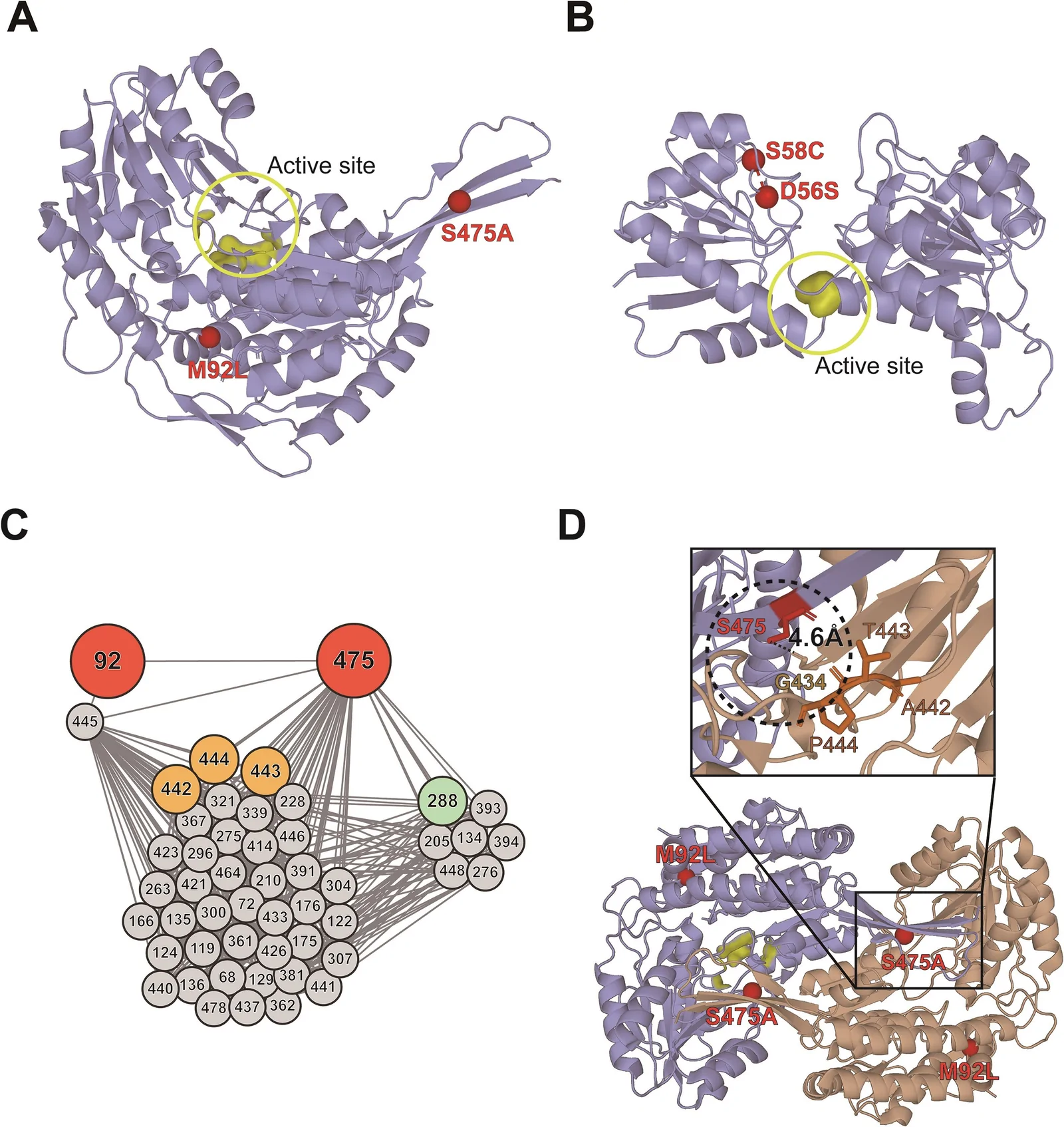
Structural and coevolutionary analysis of M92L/S475A mutations in KGSADH. (**A**) Overall structure of KGSADH showing the active site (yellow) and the structural locations of the M92L and S475A residues (red). (**B**) Overall structure of LdhA showing the active site (yellow) and the structural locations of the D56S and S58C residues (red). (**C**) Coevolution subgraph of first neighbors connected to the M92L/S475A residues. Activity-enhancing residues (92, 475; red) coevolve with functional residues (442, 443, 444; orange) and the substrate-binding residue I288 (green), revealing epistatic linkages that bridge the oligomer interface and the catalytic site. (**D**) Dimeric structure of KGSADH, highlighting functional residues and the interface distance between activity-enhancing S475 (red) from one monomer and residues from the opposing monomer (wheat). Catalytic residues critical for aldehyde binding (A442, T443, P444; orange) are shown adjacent to the active site (C287, green inset).

We analyzed the structural positioning of activity-enhancing mutations and their corresponding contributions to enzyme kinetics. To independently evaluate the catalytic performance of the previously engineered variants that successfully increased production titers, we conducted *in vitro* enzyme assays under conditions decoupled from the cellular environment (Table 1 and Supplementary Fig. S1). All variants exhibited enhanced catalytic efficiencies compared to their wild-type counterparts, showing up to 1.51- and 1.84-fold increases for KGSADH and LdhA, respectively. In the case of KGSADH, the mutants G135A/S475A and M92L/S475A showed negligible changes in the substrate-binding affinity (*K*_m_), while the turnover rate (*k*_cat_) increased substantially. These variants were positioned at sites distant from the active site in the three-dimensional structure (G135: 47.5 Å, S475: 32.8 Å, M92: 18.7 Å), all positioned outside the second shell surrounding the active site (11.9 Å) (Fig. 4A and Supplementary Fig. S4A). These findings suggest that alterations in these residues likely affected long-range interactions within the enzyme, leading to the observed increases in *k*_cat_ and overall catalytic efficiency. Similarly, the LdhA variants D56S/S58C and L121I/C173A were found outside the active-site second shell (Fig. 4B and Supplementary Fig. S4B; D56: 20.3 Å, S58: 23.8 Å, L121: 22.1 Å, C173: 24.8 Å, second shell: 7.8 Å). Furthermore, the improved catalytic efficiency of these mutants was accompanied by proportional increases in production titers, demonstrating that the epiSCANEER-predicted variants were effective in both *in vivo* and *in vitro* contexts.

**Table 1.** Kinetic characterization of superior double-site mutants in KGSADH and LdhA

Variation	*K*_m_ (mM)	Relative *k*_cat_	Relative *k*_cat_/*K*_m_
KGSADH			
WT	0.89 (±0.11)	1.00 (±0.16)	1.00 (±0.12)
G135A/S475A	1.01 (±0.15)	1.38 (±0.21)	1.22 (±0.03)
M92L/S475A	0.94 (±0.27)	1.52 (±0.36)	1.51 (±0.09)
LdhA			
WT	0.087 (±0.023)	1.00 (±0.20)	1.00 (±0.09)
L121I/C173A	0.047 (±0.014)	0.82 (±0.083)	1.55 (±0.36)
D56S/S58C	0.10 (±0.036)	2.08 (±0.49)	1.84 (±0.20)

*K*_m_ (Michaelis constant), relative *k*_cat_ (turnover number), and relative *k*_cat_/*K*_m_ (catalytic efficiency) were determined for wild-type (WT) and selected double-site variants. All values represent the mean of biological replicates (*n* = 3) with standard deviations shown in parentheses.

### Structural interpretation of the predicted mutations in the coevolution-guided model

We found that the amino acid combinations proposed by our model were difficult to predict through conventional evolution-based approaches that rely solely on individual mutation frequencies. To validate this observation, we analyzed the amino acid frequency distributions of each recommended residue position within the MSA (Supplementary Fig. S5). In KGSADH, residue 475 showed the highest frequency of phenylalanine (F), which was more prevalent than the wild-type residue serine (S). Similarly, residue 92 showed the highest frequency for methionine (M), corresponding to the query sequence. However, when pairwise amino acid combinations were considered, the model-suggested pair M92L/S475A displayed the highest evolutionary co-occurrence among all observed variants, indicating strong evolutionary compatibility. A comparable pattern was observed for LdhA. Although the most frequent non-wild-type amino acids appearing at individual positions were glutamic acid (E) and aspartic acid (D), the double-residue combination D56S/S58C exhibited the highest evolutionary frequency when pairwise occurrence was evaluated. These results demonstrate that our mutation-discovery approach, which explicitly accounts for epistatic interactions between coevolving residues, effectively identifies mutation combinations that are evolutionarily favored and functionally cooperative.

To infer the functional implications of the mutation pairs proposed by our model, we analyzed KGSADH, a well-characterized enzyme with extensive prior mutational and structural studies (Fig. 4C). Examination of the subgraph derived from the coevolution network revealed that residues M92/S475 formed strong coevolutionary connections with residues A442, T443, and P444. Previous studies have shown that these residues are located near the catalytic residue C287 and interact with the aldehyde substrate [43]. They are essential for maintaining the enzyme’s structural stability and catalytic integrity, yet exhibit strong resistance to direct mutational changes. Considering these findings, our model likely achieved positive modulation of enzymatic function not by directly altering these essential residues, but by introducing mutations into coevolving residues that are indirectly connected to them. Notably, residues M92/S475 were also found to coevolve with the substrate-binding residue I288, further supporting their potential influence on catalytic processes [44].

Furthermore, the mutations proposed in this study are likely to directly influence oligomer formation and intersubunit interactions. KGSADH requires a tetrameric (quaternary) structure for enzymatic activity, and analysis of the reported dimeric crystal structure revealed that residue 475 is positioned at the oligomeric interface, in close proximity to another monomer (4.6 Å) (Fig. 4D). Notably, several residues coevolving with residue 475 are spatially distant in the monomeric state but become adjacent upon oligomerization. This observation suggests that the coevolution network of KGSADH accurately reflects the functional and structural relationships present at the oligomeric interface. Meanwhile, substitution of serine with alanine reduces the side-chain volume, thereby forming a small cavity that induces subtle rearrangements of neighboring subunits and alters the binding mode and interfacial geometry [45]. In addition, replacement with the more hydrophobic alanine increases local hydrophobicity and promotes the exclusion of water molecules from the interface, thereby strengthening hydrophobic packing at the tetrameric interface and enhancing intersubunit stability [46].

## Discussion

In this study, we developed epiSCANEER, a computational model that prioritizes evolutionarily compatible residue pairs at double-site positions by incorporating coevolutionary dependencies. Experimental validation of mutation pairs confirmed that the residues identified by epiSCANEER achieved a 76% success rate in predicting activity-enhancing mutants, representing a 2.5-fold improvement over the conventional single-site prediction model (30%). Furthermore, structural analysis and co-evolutionary network examination of the highest-performing variants revealed that epiSCANEER successfully identified double-site mutations positioned at distal sites from the catalytic site, which modulate enzyme function through long-range interactions and oligomeric interface stabilization.

epiSCANEER stands out from existing models by explicitly accounting for epistatic interactions, thereby narrowing the search space to variants more likely to exhibit activity-enhancing effects. Non-additive interactions between residues remain a major bottleneck in mutation design, with most studies failing to adequately address them [25, 47, 48]. In our model, we directly model these epistatic relationships to identify mutations that are elusive to single-site combinatorial approaches. For example, among the mutations identified by our model in KGSADH (M92L/S475A), residue 475 received a relative titer of only 1.00 from the SCANEER model—statistically indistinguishable from wild-type and thus highly unlikely to be selected as a double-mutation candidate. However, when paired with M92L as predicted by epiSCANEER, this combination delivered a remarkable 1.50-fold titer improvement, demonstrating profound epistasis that emerges exclusively in specific residue pairs.​ By leveraging evolutionarily coupled co-evolutionary dependencies consistent with epistatic interactions, epiSCANEER expands the accessible sequence space beyond simple single-site combinations, systematically filtering out loss-of-function variants while prioritizing combinations with superior activity. Importantly, kinetic characterization indicated that these improvements were driven mainly by enhanced turnover rate in most variants, whereas substrate affinity was altered more selectively, suggesting that epiSCANEER-predicted epistatic pairs can tune distinct catalytic steps depending on structural context. This epistasis-aware approach fundamentally overcomes the limitations of laboratory evolution, positioning epiSCANEER as a transformative tool that unlocks previously inaccessible high-performance enzyme variants for protein engineering.

A limitation of epiSCANEER is its focus on double mutations, which balances statistical reliability and practical use. As more mutations are added, both the search space (20*n* for *n* positions) and the required MSA coverage grow exponentially, while the frequency of amino acid combinations becomes sparser, reducing prediction confidence [49]. Our experiments successfully demonstrated this approach across different oxidoreductases with diverse structures and functions. While testing additional enzyme types would better confirm its broad applicability, the success in detecting epistatic effects at distant sites and identifying mutations that single-site methods overlook suggest that the coevolution-based principle applies beyond these initial cases. Still, our experimental validation shows that our coevolution-based hypothesis meaningfully influences enzyme activity. Building on this, we are developing an extended framework to predict higher-order mutations. In preliminary studies, we implemented a coevolution-based triple-mutation prediction framework and experimentally evaluated predicted variants in both KGSADH and LdhA. Specifically, for KGSADH, the triple-mutation model achieved a success rate of ~60%, which was lower than that of the double-mutation model (Supplementary Table S11). For LdhA, the majority of predicted candidates retained the wild-type residue at one or more positions, rendering them largely indistinguishable from single- or double-site predictions. While these results support the feasibility of extending epiSCANEER beyond double mutations, the number of residue triplets sharing sufficiently strong coevolutionary relationships was substantially smaller than that of coevolving residue pairs, resulting in a more restricted candidate search space. Moreover, since epiSCANEER targets global regulatory sites that modulate enzyme turnover rates, it holds substantial promise for integration with mutations recommended by substrate-binding or stability-optimization models [50]. By identifying epistatic compatibility between these predictions, our approach can reveal synergistic mutation combinations that remain hidden to individual models, thereby expanding the accessible sequence space for superior enzyme variants.

To further explore the applicability of epiSCANEER beyond soluble enzymes, we applied the model to TetA, a membrane-bound tetracycline efflux protein, and experimentally evaluated the predicted double mutations by measuring specific growth rates under tetracycline selection pressure (Supplementary Table S12). While epiSCANEER successfully identified evolutionarily favorable double mutations even for this membrane protein, the success rate was markedly lower than that observed for soluble dehydrogenases. Of the 20 double mutants tested, only 2 variants exhibited higher specific growth rates than wild-type TetA under 5 µg/ml tetracycline, in contrast to the 76% success rate achieved for soluble enzymes. We attribute this reduced performance to two principal factors. First, the number of available TetA homologs in the MSA (57 sequences) was substantially smaller than those used for KGSADH (250 sequences) and LdhA (249 sequences), limiting the statistical power of co-evolutionary analysis and constraining the model’s ability to identify reliable epistatic signals. Second, and more fundamentally, epiSCANEER is based solely on residue co-evolutionary patterns derived from MSA and does not account for interactions between the protein and its surrounding molecular environment—including water, lipid bilayer, or other membrane-associated molecules—factors that critically govern the structure and function of membrane proteins. These findings delineate an important boundary condition for the current model; nevertheless, the identification of two TetA variants with improved specific growth rates under tetracycline selection demonstrates that co-evolutionary signals retain meaningful predictive power even for membrane proteins, highlighting the significance to reflect the interactions with other molecules as a key direction for future model development.

Finally, the epiSCANEER model has been deployed as a user-friendly web platform, enabling experimental biologists without computational expertise to readily access and apply our predictions. With an intuitive interface requiring only a protein sequence or MSA input, the platform delivers an end-to-end pipeline—from coevolutionary analysis and SCI scoring to top-ranked mutation recommendations—independent of library construction and screening processes. This approach effectively bridges the gap between computational prediction and wet-lab validation, empowering researchers to confidently pursue high-risk, high-reward mutation combinations. Ultimately, it accelerates iterative design-build-test-learn (DBTL) workflows and is poised to substantially enhance productivity across enzyme engineering research.

## Supplementary Material

gkag879_Supplemental_File

## Data Availability

All experimental data supporting the findings of this study are available within the article and its Supplementary data files. Code availability Extensions of our method are possible, including the engineering of any desired enzymes via our web server (https://sbiweb.postech.ac.kr:3051/w/epiSCANEER/). The source code, SCI calculation scripts, and example datasets used in this study are available at https://github.com/khsub87/epiSCANEER and Zenodo at https://doi.org/10.5281/zenodo.22092800.

## Notes

Present address: Synthetic Biology Research Center, Korea Research Institute of Bioscience and Biotechnology (KRIBB), Daejeon, 34141, Korea
