# Supplementary material for: Enhancing prediction accuracy for enzyme activity engineering through sequence co-evolution and epistatic relationship modeling
Source: Nucleic Acids Res. 2026 Sep 8;54(17):gkag879. doi: 10.1093/nar/gkag879 (PMC13551031; doi:10.1093/nar/gkag879)
Supplement: Supplementary file 1 [file gkag879_supplemental_file.docx]

**Enhancing prediction accuracy for enzyme activity engineering through sequence co-evolution and epistatic relationship modeling**

**Supplementary data**

Da Neub Kim^1,2,*^, Hyeongseop Kim^1,*^, Donghyo Kim^3^, Gyoo Yeol Jung^1,3,✝^, Sanguk Kim^4,✝^, Myung Hyun Noh^2,a✝^

^1^School of Interdisciplinary Bioscience and Bioengineering, Pohang University of Science and Technology, Pohang, Gyeongbuk, Korea

^2^Center for Bio-based Chemistry, Korea Research Institute of Chemical Technology, Ulsan, Korea

^3^Department of Chemical Engineering, Pohang University of Science and Technology, Pohang, Gyeongbuk, Korea

^4^Department of Life Sciences, Pohang University of Science and Technology, Pohang, Gyeongbuk, Korea

^a^Current Affiliation: Synthetic Biology Research Center, Korea Research Institute of Bioscience and Biotechnology (KRIBB), Daejeon, Korea

*These authors contributed equally to this work.

†To whom correspondence should be addressed.

(M. H. Noh) : Synthetic Biology Research Center, Korea Research Institute of Bioscience and Biotechnology (KRIBB), 125, Gwahak-ro, Yuseong-gu, Daejeon, 34141, South Korea

Tel.: +82 42 860 4375; Email: mhnoh@kribb.re.kr

(S. U. Kim) E-mail: sukim@postech.ac.kr

(G. Y. Jung) E-mail: [gyjung@postech.ac.kr](mailto:gyjung@postech.ac.kr)

**Supplementary Figures**

**Supplementary Figure S1.** **SDS-PAGE analysis of purified His-tagged enzymes.** SDS-PAGE analysis of purified **(A)** KGSADH and **(B)** LdhA showing wild-type and superior double-site mutants. 'L' represents the molecular weight marker (PageRuler^TM^ Prestained Protein Ladder, Thermo Scientific). Expected molecular weights based on protein sequences: KGSADH, 52.1 kDa; LdhA, 37.58 kDa.

**Supplementary Figure S2. SCI score and relative titer across ranked groups for KGSADH (A, B) and LdhA (C, D). (A, C)** Average relative titer of five variants within each SCI quartile (Q1–Q4), where Q1 corresponds to ranked groups G1–G5 (lowest SCI) and Q4 corresponds to ranked groups G16–G20 (highest SCI). Bars represent the mean relative titer of five representative variants per quartile. Error bars represent the standard error of the mean (SEM, *n* = 5). **(B, D)** Individual titer measurements of representative variants within the highest-ranked groups (G16–G20), where each ranked group (G) represents a distinct SCI score interval within the top quartile. Each dot represents the mean relative titer of biological triplicates (*n* = 3) with standard deviation.

**Supplementary Figure S3. Comparison of SCI score distributions between all possible amino acid combinations at double-residue positions and selected combinations.** **(A)** Residue pair 92/475 in KGSADH. **(B)** Residue pair 56/58 in LdhA. Graphs show probability density (y-axis) versus SCI score (x-axis) for all possible combinations at both positions (skyblue bars) and combinations obtained by fixing one position's amino acid while varying the other (orange bars).

**Supplementary Figure S4.** **Structural location of additional activity-enhancing mutations in KGSADH and LdhA.** **(A)** Overall structure of KGSADH showing the active site (yellow) and the structural locations of the G135A and S475A residues (red). **(B)** Overall structure of LdhA showing the active site (yellow) and the structural locations of the L121I and C173A residues (red).

**Supplementary Figure S5. Amino acid frequency distributions for individual residues and residue pairs derived from the multiple sequence alignment (MSA).** In all graphs, black bars indicate wild-type amino acids, gray bars represent single mutations, blue bars denote amino acids predicted by epiSCANEER, and light-blue bars correspond to double mutations within residue pairs. **(A–C)** Frequency distributions for residues 92 and 475 in KGSADH: **(A)** amino acid pair frequency between positions 92 and 475, **(B)** amino acid frequency at position 92, and **(C)** amino acid frequency at position 475. **(D–F)** Corresponding distributions for residues 56 and 58 in LdhA: **(D)** amino acid pair frequency, **(E)** position 56, and **(F)** position 58.

**Supplementary Tables**

| **Strains** | **Description** | **Source** |
| --- | --- | --- |
| *E. coli* DH5α | Expression host for TetA, Cloning host | BioFACT |
| *E. coli* W | Expression host for KGSADH, an acid-tolerant *E. coli* strain | ATCC9637 |
| *Vibrio* sp. dhg | Expression host for LdhA, a halophilic and fast-growing *Vibrio* strain | (1) |
| VΔ5 | *Vibrio* sp. dhg Δ*dns* Δ*ldhA* Δ*frdABCD* Δ*pflB* Δ*ack-pta* | (1) |
| WBK | *E. coli* W / pACYC_B4 / pQE80L-K (or pQE80L-K^mut^) | In this study |
| VL | VΔ5 / pACYC_ldhA (or pACYC_ldhA^mut^) | In this study |
| DT | *E. coli* DH5α / pACYC_J23113_tetA (or pACYC_J23113_tetA^mut^) | In this study |
| **Plasmids** | **Description** | **Source** |
| pACYC_B4 | p15A ori, Cm^R^, P_tac__synUTR4_dhaB1__*dhaB1*_P_tac__synUTR_dhaB2__*dhaB2*_*dhaB3*_*gdrA*_*gdrB* | (2) |
| pQE80L-K | ColE1 ori, Amp^R^, P_T5__6X His_*kgsadh* | (2) |
| pQE80L-K^mut^ | ColE1 ori, Amp^R^, P_T5__6X His_*kgsadh*^mut^ | In this study |
| pACYC_galP_ldhA | p15A ori, Cm^R^, P_J23100__synUTR_*galP*_BBa_B1001 terminator, P_J23100__synUTR_*ldhA*_BBa_B1001 terminator | (3) |
| pACYC_ldhA | p15A ori, Cm^R^, P_J23100__synUTR_*ldhA*_6X His_BBa_B1001 terminator | In this study |
| pACYC_ldhA^mut^ | p15A ori, Cm^R^, P_J23100__synUTR_*ldhA*^mut^_6X His_BBa_B1001 terminator | In this study |
| pCDF-3HPselector | CloDF13 ori, Sm^R^, P_J23106__synUTR_C4lysR_-*C4lysR*_terminator-P_C4M__synUTR_tetA_-*tetA* | (2) |
| pACYC_J23113_tetA | p15A ori, Cm^R^, P_J23113__*tetA*_T7 terminator | In this study |
| pACYC_J23113_tetA^mut^ | p15A ori, Cm^R^, P_J23113__*tetA*^mut^_T7 terminator | In this study |

**Supplementary Table S1. Strains and plasmids used in this study.** Amp, ampicillin; Cm, chloramphenicol; R, resistance; ATCC, American Type Culture Collection

| **Protein** | **Protein sequence** | **Reference** |
| --- | --- | --- |
| KGSADH  (alpha-ketoglutaric semialdehyde dehydrogenase, UniProtKB: Q1JUP4) | MRGSHHHHHHGSANVTYTDTQLLIDGEWVDAASGKTIDVVNPATGKPIGRVAHAGIADLDRALAAAQSGFEAWRKVPAHERAATMRKAAALVRERADAIAQLMTQEQGKPLTEARVEVLSAADIIEWFADEGRRVYGRIVPPRNLGAQQTVVKEPVGPVAAFTPWNFPVNQVVRKLSAALATGCSFLVKAPEETPASPAALLRAFVDAGVPAGVIGLVYGDPAEISSYLIPHPVIRKVTFTGSTPVGKQLASLAGLHMKRATMELGGHAPVIVAEDADVALAVKAAGGAKFRNAGQVCISPTRFLVHNSIRDEFTRALVKHAEGLKVGNGLEEGTTLGALANPRRLTAMASVIDNARKVGASIETGGERIGSEGNFFAPTVIANVPLDADVFNNEPFGPVAAIRGFDKLEEAIAEANRLPFGLAGYAFTRSFANVHLLTQRLEVGMLWINQPATPWPEMPFGGVKDSGYGSEGGPEALEPYLVTKSVTVMAV* | (2) |
| LdhA  (lactate dehydrogenase A, UniParc: UPI000E531CA4) | MINIAFFSAKSYDEASFNKVKNQRDLEFHYHDFRLTTKTAKMAHGCEVVCAFVNDDLSEPVLKQLSQGGTKLIAMRCAGFDKVDQQAAKKLGLQVVHVPAYSPEAVAEHTVGMMMCLNRRLHKAYQRTRDANFSLEGLVGFNFFGKTVGVIGTGKIGIAAMRIFKGLGMEILCHDPYENPLAIEMGARYCSLEDIYANADIITLHCPMSKENYHLLNADSFSKMKDGVMIINTSRGELLDSVAAIEALKQGRIGSLGLDVYDNEKELFFQDKSNDIIVDDVFRRLSACHNVLFTGHQAFLTHEALNNIASVTLNNVEVFFSGQVSGNELINHHHHHH* | (3) |
| TetA  (tetracycline resistance structural protein TetA, UniProtKB: Q79SH9) | MKSNNALIVILGTVTLDAVGIGLVMPVLPGLLRDIVHSDSIASHYGVLLALYALMQFLCAPVLGALSDRFGRRPVLLASLLGATIDYAIMATTPVLWILYAGRIVAGITGATGAVAGAYIADITDGEDRARHFGLMSACFGVGMVAGPVAGGLLGAISLHAPFLAAAVLNGLNLLLGCFLMQESHKGERRPMPLRAFNPVSSFRWARGMTIVAALMTVFFIMQLVGQVPAALWVIFGEDRFRWSATMIGLSLAVFGILHALAQAFVTGPATKRFGEKQAIIAGMAADALGYVLLAFATRGWMAFPIMILLASGGIGMPALQAMLSRQVDDDHQGQLQGSLAALTSLTSIIGPLIVTAIYAASASTWNGLAWIVGAALYLVCLPALRRGAWSRATST* | (2) |

**Supplementary Table S2. Wild-type amino acid sequences of KGSADH, LdhA, and TetA used in this study.** The 6×His-tag sequence appended for protein purification is indicated in red.

| **Quarter** | **Ranked**  **group** | **Variation** | **SCI** | **Titer fold**  **(mean ±error)** | ***p*-value** |
| --- | --- | --- | --- | --- | --- |
| Q1 | G1 | P441Y/V478L | 0.282 | 1.166 ± 0.018 | 2.2.E-03 |
|  | G2 | Q440T/V478L | 0.369 | 0.446 ± 0.027 | 3.0.E-05 |
|  | G3 | G205S/V478T | 0.416 | 0.818 ± 0.412 | ns |
|  | G4 | Q137A/P441M | 0.451 | - | - |
|  | G5 | H68L/S475I | 0.475 | 1.113 ± 0.020 | 9.5.E-03 |
| Q2 | G6 | V124S/N423Y | 0.500 | 0.746 ± 0.018 | 4.3.E-04 |
|  | G7 | L270F/P464C | 0.524 | 1.032 ± 0.037 | ns |
|  | G8 | A136H/Q440S | 0.546 | 1.121 ± 0.013 | 5.8.E-03 |
|  | G9 | V124A/L270R | 0.569 | 0.299 ± 0.016 | 7.0.E-06 |
|  | G10 | H68Y/V433I | 0.600 | 0.292 ± 0.020 | 7.9.E-06 |
| Q3 | G11 | D49A/E95D | 0.625 | 0.787 ± 0.020 | 9.0.E-04 |
|  | G12 | A275S/W445V | 0.650 | - | - |
|  | G13 | L134P/V433L | 0.680 | 1.132 ± 0.112 | ns |
|  | G14 | A72V/A367E | 0.710 | 0.425 ± 0.035 | 4.0.E-05 |
|  | G15 | V162C/K226L | 0.736 | - | - |
| Q4 | G16 | R127Q/S460D | 0.756 | - | - |
|  | G17 | L326I/D379K | 0.781 | 0.984 ± 0.019 | ns |
|  | G18 | V18I/D379K | 0.805 | 1.155 ± 0.010 | 2.1.E-03 |
|  | G19 | G414A/V478I | 0.830 | 1.184 ± 0.005 | 1.0.E-03 |
|  | G20 | L12H/V195L | 0.853 | 1.222 ± 0.013 | 5.9.E-04 |

**Supplementary Table S3. Enzyme activity validation of KGSADH mutants predicted by epiSCANEER across SCI—ranked group.** G1–G20 indicate ranked groups sorted in ascending order of SCI score, where G20 corresponds to the top 5^th^ percentile. Titer fold-change indicates the mean relative product concentration compared to wild-type. '-' denotes loss-of-function variants for which titer was not detected. All data represent biological replicates (*n* = 3) with standard deviations. *p*-values indicate the statistical significance of titer fold-change measured by two-tailed Student's *t*-test, where "ns" denotes not significant (*P* > 0.05).

| **Quarter** | **Ranked**  **group** | **Variation** | **SCI** | **Titer fold**  **(mean ±error)** | ***p*-value** |
| --- | --- | --- | --- | --- | --- |
| Q1 | G1 | M114A/D275Y | 0.122 | 0.623 ± 0.033 | 1.5.E-03 |
|  | G2 | D32Q/D275R | 0.183 | 0.761 ± 0.059 | 1.3.E-02 |
|  | G3 | A131S/C288F | 0.226 | - | - |
|  | G4 | D32T/V228S | 0.265 | - | - |
|  | G5 | K155Q/F293M | 0.301 | 1.036 ± 0.015 | ns |
| Q2 | G6 | Q126N/F293M | 0.335 | 1.045 ± 0.008 | ns |
|  | G7 | M114L/F293I | 0.370 | 0.875 ± 0.009 | ns |
|  | G8 | K155A/K265M | 0.401 | 0.989 ± 0.065 | ns |
|  | G9 | K41L/A244I | 0.430 | 0.531 ± 0.125 | 5.3.E-03 |
|  | G10 | M169C/D275I | 0.456 | 0.152 ± 0.016 | 5.1.E-05 |
| Q3 | G11 | A182F/F221I | 0.473 | 0.878 ± 0.026 | ns |
|  | G12 | L216I/N263A | 0.496 | 0.763 ± 0.081 | 2.2.E-02 |
|  | G13 | H31L/V228S | 0.529 | - | - |
|  | G14 | K41P/C288F | 0.558 | 1.085 ± 0.074 | ns |
|  | G15 | F33P/K265R | 0.594 | 1.037 ± 0.042 | ns |
| Q4 | G16 | M161A/I230L | 0.648 | 0.924 ± 0.065 | ns |
|  | G17 | K165H/I276M | 0.712 | 0.793 ± 0.071 | 2.7.E-02 |
|  | G18 | Y177A/L313M | 0.790 | 1.034 ± 0.028 | ns |
|  | G19 | N118S/H205Y | 0.855 | 0.623 ± 0.164 | 2.3.E-02 |
|  | G20 | K21D/R24E | 0.877 | 1.216 ± 0.043 | 1.4.E-02 |

**Supplementary Table S4. Enzyme activity validation of LdhA mutants predicted by epiSCANEER across SCI—ranked group.** G1–G20 indicate ranked groups sorted in ascending order of SCI score, where G20 corresponds to the top 5^th^ percentile. Titer fold-change indicates the mean relative product concentration compared to wild-type. '-' denotes loss-of-function variants for which titer was not detected. All data represent biological replicates (*n* = 3) with standard deviations. *p*-values indicate the statistical significance of titer fold-change measured by two-tailed Student's *t*-test, where "ns" denotes not significant (*P* > 0.05).

| **Variation** | **SCI^*^** | **Titer fold**  **(mean ±error)** | ***p*-value** |
| --- | --- | --- | --- |
| A328P | 2.459 | 1.152 ± 0.048 | 3.6.E-03 |
| G135A | 1.570 | 1.022 ± 0.021 | 2.8.E-02 |
| T139L | 1.568 | 1.029 ± 0.039 | 4.4.E-02 |
| T139M | 1.423 | 0.974 ± 0.082 | ns |
| P469A | 1.089 | 0.845 ± 0.023 | 1.9.E-02 |
| I371L | 1.077 | 0.939 ± 0.009 | ns |
| N373D | 0.974 | 0.935 ± 0.078 | ns |
| W445S | 0.719 | 1.130 ± 0.015 | 1.1.E-03 |
| G135N | 0.590 | 0.438 ± 0.016 | 2.5.E-05 |
| S475A | 0.562 | 1.002 ± 0.024 | ns |
| F421L | 0.547 | 1.034 ± 0.005 | 1.1.E-02 |
| G135S | 0.532 | 1.047 ± 0.025 | 1.4.E-02 |
| S109A | 0.500 | 1.091 ± 0.068 | 2.7.E-02 |
| A189E | 0.493 | 1.002 ± 0.032 | ns |
| T139I | 0.463 | 0.532 ± 0.012 | 5.0.E-05 |
| V18C | 0.406 | 1.505 ± 0.007 | 1.2.E-05 |
| T304A | 0.304 | 0.449 ± 0.003 | 2.0.E-05 |
| E362A | 0.302 | 0.923 ± 0.006 | ns |
| T477S | 0.294 | 1.019 ± 0.009 | 2.2.E-02 |
| E321A | 0.224 | 1.014 ± 0.008 | 3.7.E-02 |
| I342V | 0.137 | 0.991 ± 0.007 | ns |
| R192Q | 0.064 | 1.051 ± 0.032 | 1.6.E-02 |
| G135D | 0.009 | 1.005 ± 0.018 | 4.9.E-02 |

^*^SCI values were calculated using the original SCANEER scoring formula.

**Supplementary Table S5. Enzyme activity validation of KGSADH mutants predicted by SCANEER.** Titer fold-change indicates the mean relative product concentration compared to wild-type. All data represent biological replicates (*n* = 3) with standard deviations. *p*-values indicate the statistical significance of titer fold-change measured by two-tailed Student's *t*-test, where "ns" denotes not significant (*P* > 0.05).

| **Variation** | **SCI** | **Titer fold**  **(mean ±error)** | ***p*-value** |
| --- | --- | --- | --- |
| W445S/S475A | 1.000 | 1.090 ± 0.044 | 3.0E-02 |
| G135A/W445S | 0.971 | 1.047 ± 0.159 | ns |
| G135A/S475A | 0.959 | 1.336 ± 0.012 | 1.0E-05 |
| F421L/W445S | 0.959 | 1.101 ± 0.016 | 1.8E-03 |
| F421L/S475A | 0.947 | 1.087 ± 0.108 | ns |
| T304A/W445S | 0.938 | 0.848 ± 0.059 | 1.3E-02 |
| E362A/W445S | 0.937 | 1.186 ± 0.012 | 1.0E-04 |
| E321A/W445S | 0.936 | 1.103 ± 0.044 | 2.0E-02 |
| W445S/V478I | 0.934 | 0.981 ± 0.049 | ns |
| S166G/W445S | 0.934 | 1.182 ± 0.029 | 7.6E-04 |
| M92L/W445S | 0.933 | 1.239 ± 0.025 | 1.6E-04 |
| T304A/S475A | 0.928 | 0.810 ± 0.097 | 2.9E-02 |
| E362A/S475A | 0.927 | 1.102 ± 0.029 | 6.3E-03 |
| E321A/S475A | 0.926 | 1.061 ± 0.010 | 5.9E-03 |
| M92L/S475A | 0.925 | 1.499 ± 0.012 | 2.2E-05 |
| S475A/V478I | 0.924 | 1.111 ± 0.039 | 1.1E-02 |
| A328P/I371L | 0.917 | 1.152 ± 0.049 | 7.3E-03 |
| G135A/V478I | 0.895 | 1.030 ± 0.039 | ns |
| F421L/V478I | 0.888 | 0.999 ± 0.037 | ns |
| K35Q/E362A | 0.866 | 0.638 ± 0.075 | 1.3E-03 |
| V158I/V424A | 0.862 | 1.142 ± 0.051 | 1.0E-02 |

**Supplementary Table S6. Enzyme activity validation of KGSADH mutants predicted by epiSCANEER.** Titer fold-change indicates the mean relative product concentration compared to wild-type. All data represent biological replicates (*n* = 3) with standard deviations. *p*-values indicate the statistical significance of titer fold-change measured by two-tailed Student's *t*-test, where "ns" denotes not significant (*P* > 0.05).

| **Variation** | **SCI** | **Titer fold**  **(mean ±error)** | ***p*-value** |
| --- | --- | --- | --- |
| H31F/I276V | 1.000 | 0.121 ± 0.096 | 2.7E-04 |
| K165R/I276V | 0.938 | 1.349 ± 0.225 | ns |
| N3K/H31F | 0.923 | 1.515 ± 0.044 | 6.6E-04 |
| H31F/K82N | 0.918 | 1.330 ± 0.140 | 2.4E-02 |
| A5G/I276V | 0.914 | 0.739 ± 0.307 | ns |
| N142T/A160T | 0.908 | - | - |
| S255A/K265R | 0.908 | 0.473 ± 0.306 | 4.5E-02 |
| E14R/H97R | 0.891 | - | - |
| K21N/R24F | 0.887 | 0.798 ± 0.162 | ns |
| L121I/C173A | 0.885 | 1.684 ± 0.401 | 2.8E-03 |
| I253L/I276V | 0.884 | 1.126 ± 0.395 | ns |
| I2M/V278Q | 0.883 | 1.340 ± 0.087 | 7.9E-03 |
| I2M/D262E | 0.879 | 1.268 ± 0.031 | 6.1E-03 |
| M161L/K265R | 0.879 | 0.728 ± 0.486 | ns |
| D240N/A243D | 0.878 | 1.208 ± 0.032 | 1.5E-02 |
| I2L/N3D | 0.876 | 1.341 ± 0.066 | 5.0E-03 |
| D56S/S58C | 0.876 | 1.773 ± 0.054 | 1.7E-04 |
| A74L/G257A | 0.872 | 1.386 ± 0.065 | 3.1E-03 |
| T153C/Y189Q | 0.870 | - | - |
| E136D/I276V | 0.870 | 1.539 ± 0.127 | 3.5E-03 |

**Supplementary Table S7. Enzyme activity validation of LdhA mutants predicted by epiSCANEER.** Titer fold-change indicates the mean relative product concentration compared to wild-type. '-' denotes loss-of-function variants for which titer was not detected. All data represent biological replicates (*n* = 3) with standard deviations. *p*-values indicate the statistical significance of titer fold-change measured by two-tailed Student's *t*-test, where "ns" denotes not significant (*P* > 0.05).

| **Variation** | **SCI** | **Titer fold**  **(mean ±error)** | ***p*-value** |
| --- | --- | --- | --- |
| M92L/S475A | 0.925 | 1.499 ± 0.012 | 2.2E-05 |
| M92M/S475A | 0.922 | 1.002 ± 0.024 | ns |
| M92I/S475A | 0.914 | 1.102 ± 0.008 | 2.2E-05 |
| M92A/S475A | 0.910 | 1.352 ± 0.061 | 1.7E-03 |
| M92C/S475A | 0.910 | - | - |
| M92D/S475A | 0.910 | - | - |
| M92E/S475A | 0.910 | 1.246 ± 0.051 | 3.4E-03 |
| M92F/S475A | 0.910 | - | - |
| M92G/S475A | 0.910 | - | - |
| M92H/S475A | 0.910 | - | - |
| M92K/S475A | 0.910 | - | - |
| M92N/S475A | 0.910 | 1.198 ± 0.073 | 2.4E-02 |
| M92P/S475A | 0.910 | - | - |
| M92Q/S475A | 0.910 | - | - |
| M92R/S475A | 0.910 | - | - |
| M92S/S475A | 0.910 | - | - |
| M92T/S475A | 0.910 | - | - |
| M92V/S475A | 0.910 | 0.760 ± 0.040 | 1.4E-03 |
| M92W/S475A | 0.910 | - | - |
| M92Y/S475A | 0.910 | - | - |
| M92L/M475S | 0.858 | 0.777 ± 0.018 | 9.7E-05 |
| M92L/S475L | 0.704 | 0.759 ± 0.014 | 3.0E-05 |
| M92L/S475T | 0.696 | 0.783 ± 0.015 | 3.5E-07 |
| M92L/S475F | 0.682 | - | - |
| M92L/S475V | 0.608 | 0.755 ± 0.009 | 5.2E-06 |
| M92L/S475Y | 0.560 | - | - |
| M92L/S475I | 0.536 | 0.732 ± 0.033 | 4.7E-04 |
| M92L/S475C | 0.524 | 0.670 ± 0.032 | 1.9E-04 |
| M92L/S475D | 0.524 | 0.635 ± 0.018 | 1.4E-05 |
| M92L/S475E | 0.524 | 0.563 ± 0.003 | 2.5E-08 |
| M92L/S475G | 0.524 | 0.835 ± 0.157 | 2.4E-01 |
| M92L/S475H | 0.524 | 0.704 ± 0.004 | 1.9E-07 |
| M92L/S475K | 0.524 | - | - |
| M92L/S475M | 0.524 | 0.786 ± 0.003 | 5.5E-05 |
| M92L/S475N | 0.524 | 0.767 ± 0.027 | 3.7E-04 |
| M92L/S475P | 0.524 | - | - |
| M92L/S475Q | 0.524 | 0.889 ± 0.164 | 4.3E-01 |
| M92L/S475R | 0.524 | - | - |
| M92L/S475W | 0.524 | - | - |

**Supplementary Table S8. Site-saturated mutagenesis of M92L/S475A residues in KGSADH predicted by epiSCANEER.** One residue of M92L/S475A was fixed while the other position was substituted with all canonical amino acids. Titer fold-change indicates the mean relative product concentration compared to wild-type. '-' denotes loss-of-function variants for which titer was not detected. All data represent biological replicates (*n* = 3) with standard deviations. *p*-values indicate the statistical significance of titer fold-change measured by two-tailed Student's *t*-test, where "ns" denotes not significant (*P* > 0.05).

| **Variation** | **SCI** | **Titer fold**  **(mean ±error)** | ***p*-value** |
| --- | --- | --- | --- |
| D56S/S58C | 0.876 | 1.773 ± 0.054 | 1.7E-04 |
| D56D/S58C | 0.873 | 0.451 ± 0.018 | 2.3E-05 |
| D58N/S58C | 0.871 | 0.333 ± 0.066 | 8.1E-04 |
| D56E/S58C | 0.871 | 0.670 ± 0.096 | 8.2E-04 |
| D56A/S58C | 0.870 | 0.300 ± 0.030 | 1.1E-03 |
| D56C/S58C | 0.870 | 0.244 ± 0.123 | 2.9E-06 |
| D56F/S58C | 0.870 | 0.538 ± 0.018 | 6.0E-03 |
| D56G/S58C | 0.870 | 0.274 ± 0.148 | 6.2E-05 |
| D56H/S58C | 0.870 | - | - |
| D56I/S58C | 0.870 | - | - |
| D56K/S58C | 0.870 | 0.226 ± 0.002 | 4.2E-06 |
| D56L/S58C | 0.870 | 0.650 ± 0.025 | 5.4E-04 |
| D56M/S58C | 0.870 | 0.257 ± 0.002 | 1.4E-05 |
| D56N/S58C | 0.870 | 0.301 ± 0.040 | 7.9E-06 |
| D56P/S58C | 0.870 | 0.562 ± 0.029 | 1.1E-03 |
| D56Q/S58C | 0.870 | 0.471 ± 0.053 | 3.8E-04 |
| D56R/S58C | 0.870 | 1.037 ± 0.037 | 2.9E-01 |
| D56T/S58C | 0.870 | 0.941 ± 0.044 | 2.5E-01 |
| D56V/S58C | 0.870 | - | - |
| D56W/S58C | 0.870 | 0.867 ± 0.003 | 4.5E-03 |
| D56Y/S58C | 0.870 | 0.991 ± 0.027 | 7.3E-01 |
| D56S/S58A | 0.870 | 0.959 ± 0.016 | 7.7E-02 |
| D56S/S58D | 0.870 | 0.453 ± 0.006 | 1.5E-03 |
| D56S/S58E | 0.870 | 0.626 ± 0.082 | 2.4E-04 |
| D56S/S58F | 0.870 | 0.850 ± 0.043 | 7.1E-03 |
| D56S/S58G | 0.870 | 0.481 ± 0.010 | 7.7E-04 |
| D56S/S58H | 0.870 | 0.496 ± 0.056 | 3.0E-05 |
| D56S/S58I | 0.870 | 0.699 ± 0.029 | 1.7E-03 |
| D56S/S58K | 0.870 | 0.504 ± 0.063 | 4.0E-04 |
| D56S/S58L | 0.870 | 0.470 ± 0.073 | 8.4E-05 |
| D56S/S58M | 0.870 | 0.649 ± 0.048 | 1.1E-03 |
| D56S/S58P | 0.870 | 0.705 ± 0.043 | 2.6E-03 |
| D56S/S58Q | 0.870 | 0.393 ± 0.100 | 5.5E-04 |
| D56S/S58R | 0.870 | 0.637 ± 0.014 | 4.2E-05 |
| D56S/S58S | 0.870 | 0.567 ± 0.055 | 2.7E-04 |
| D56S/S58T | 0.870 | 0.535 ± 0.058 | 2.5E-04 |
| D56S/S58V | 0.870 | 0.391 ± 0.041 | 2.9E-04 |
| D56S/S58W | 0.870 | 0.697 ± 0.056 | 1.2E-03 |
| D56S/S58Y | 0.870 | 0.519 ± 0.061 | 2.5E-04 |

**Supplementary Table S9. Site-saturated mutagenesis of D56S/S58C residues in LdhA predicted by epiSCANEER.** One residue of D56S/S58C was fixed while the other position was substituted with all canonical amino acids. Titer fold-change indicates the mean relative product concentration compared to wild-type. '-' denotes loss-of-function variants for which titer was not detected. All data represent biological replicates (*n* = 3) with standard deviations. *p*-values indicate the statistical significance of titer fold-change measured by two-tailed Student's *t*-test, where "ns" denotes not significant (*P* > 0.05).

| **Variation** | **Titer fold**  **(mean ±error)** | ***p*-value** |
| --- | --- | --- |
| V18C/S109A | 1.516 ± 0.012 | 1.8E-06 |
| V18C/G135S | 1.423 ± 0.021 | 1.1E-05 |
| V18C/R192Q | 1.492 ± 0.016 | 3.4E-06 |
| V18C/A328P | 1.463 ± 0.014 | 3.4E-06 |
| V18C/F421L | - | - |
| V18C/W445S | 1.414 ± 0.041 | 8.6E-05 |
| S109A/G135S | 0.896 ± 0.035 | 1.0E-02 |
| S109A/R192Q | 0.979 ± 0.015 | ns |
| S109A/A328P | 0.898 ± 0.007 | 7.1E-04 |
| S109A/F421L | 0.914 ± 0.012 | 2.0E-03 |
| S109A/W445S | 0.804 ± 0.208 | ns |
| G135S/R192Q | 0.760 ± 0.008 | 2.5E-05 |
| G135S/A328P | 1.039 ± 0.005 | 1.9E-02 |
| G135S/F421L | 0.845 ± 0.031 | 1.6E-03 |
| G135S/W445S | 1.031 ± 0.009 | 5.1E-02 |
| R192Q/A328P | 0.851 ± 0.010 | 2.1E-04 |
| R192Q/F421L | 0.977 ± 0.004 | ns |
| R192Q/W445S | 1.031 ± 0.017 | ns |
| A328P/F421L | 0.805 ± 0.017 | 1.5E-04 |
| A328P/W445S | 1.000 ± 0.027 | ns |
| F421L/W445S | 0.952 ± 0.004 | 9.8E-03 |

**Supplementary Table S10. Enzyme activity validation of KGSADH mutation combinations predicted by SCANEER.** Titer fold-change indicates the mean relative product concentration compared to wild-type. '-' denotes loss-of-function variants for which titer was not detected. All data represent biological replicates (*n* = 3) with standard deviations. *p*-values indicate the statistical significance of titer fold-change measured by two-tailed Student's *t*-test, where "ns" denotes not significant (*P* > 0.05).

| **Variation** | **SCI** | **Titer fold**  **(mean ±error)** | ***p*-value** |
| --- | --- | --- | --- |
| S475A/W445S/G135A | 1.000 | 1.251 ± 0.007 | 3.1.E-04 |
| S475A/F421L/W445S | 0.931 | 1.252 ± 0.008 | 3.1.E-04 |
| E321A/S475A/W445S | 0.809 | - | - |
| S475A/M92L/W445S | 0.806 | 1.218 ± 0.025 | 1.1.E-03 |
| F421L/W445S/G135A | 0.764 | 1.199 ± 0.004 | 7.3.E-04 |
| S475A/F421L/G135A | 0.689 | 1.172 ± 0.008 | 1.4.E-03 |
| T304A/W445S/G135A | 0.651 | 0.397 ± 0.135 | 1.7.E-03 |
| M92L/W445S/G135A | 0.625 | - | - |
| F421L/V478I/W445S | 0.593 | 1.033 ± 0.009 | ns |
| T304A/W445S/F421L | 0.582 | 0.810 ± 0.017 | 1.3.E-03 |
| T304A/S475A/G135A | 0.580 | 0.646 ± 0.027 | 1.8.E-04 |
| S475A/M92L/G135A | 0.567 | 1.235 ± 0.012 | 4.6.E-04 |
| S475A/F421L/V478I | 0.528 | 1.330 ± 0.022 | 1.8.E-04 |
| S475A/M92L/F421L | 0.505 | 1.282 ± 0.013 | 2.3.E-04 |
| T304A/W445S/V478I | 0.447 | - | - |
| T304A/W445S/S166G | 0.446 | 0.750 ± 0.015 | 4.0.E-04 |
| E362A/W445S/S166G | 0.439 | 0.420 ± 0.018 | 1.7.E-05 |
| E321A/W445S/V478I | 0.437 | 1.103 ± 0.025 | 1.6.E-02 |
| E362A/M92L/W445S | 0.436 | 1.234 ± 0.011 | 4.5.E-04 |
| E321A/W445S/S166G | 0.434 | 1.233 ± 0.028 | 9.5.E-04 |

**Supplementary Table S11. Enzyme activity validation of higher-order mutation combinations in KGSADH predicted by epiSCANEER.** Titer fold-change indicates the mean relative product concentration compared to wild-type. '-' denotes loss-of-function variants for which titer was not detected. All data represent biological replicates (*n* = 3) with standard deviations. *p*-values indicate the statistical significance of titer fold-change measured by two-tailed Student's *t*-test, where "ns" denotes not significant (*P* > 0.05).

| **Variation** | **SCI** | **Specific growth rate fold**  **(mean ±error)** | ***p*-value** |
| --- | --- | --- | --- |
| M181L/A214P | 1.000 | 0.361 ± 0.111 | 1.7.E-03 |
| M181L/S312L | 0.971 | 0.512 ± 0.148 | 8.7.E-03 |
| M181L/I308P | 0.962 | 0.227 ± 0.035 | 2.0.E-04 |
| M181L/Q182P | 0.961 | 0.536 ± 0.021 | 1.2.E-03 |
| A214P/S312L | 0.832 | 0.376 ± 0.060 | 6.8.E-04 |
| A214P/I308P | 0.818 | 0.422 ± 0.028 | 5.6.E-04 |
| M144F/A214P | 0.804 | 0.480 ± 0.078 | 1.9.E-03 |
| I308P/S312L | 0.785 | 1.080 ± 0.108 | ns |
| M144F/S312L | 0.771 | 0.616 ± 0.059 | 4.2.E-03 |
| M144F/I308P | 0.764 | 0.297 ± 0.079 | 6.2.E-04 |
| M144F/Q182P | 0.750 | 0.410 ± 0.059 | 8.4.E-04 |
| Q182P/F197L | 0.731 | 0.783 ± 0.072 | 3.5.E-02 |
| Q182P/F203L | 0.725 | 0.679 ± 0.077 | 1.1.E-02 |
| I35L/F203L | 0.718 | 0.309 ± 0.109 | 1.2.E-03 |
| H132R/F203L | 0.715 | 0.391 ± 0.040 | 5.4.E-04 |
| M247T/A270L | 0.709 | 1.225 ± 0.079 | 3.5.E-02 |
| A156G/A270L | 0.703 | 1.392 ± 0.041 | 2.9.E-03 |
| A270L/S362T | 0.692 | 0.480 ± 0.106 | 3.3.E-03 |
| I35L/T347A | 0.691 | 1.193 ± 0.124 | ns |
| I35L/T93A | 0.688 | 0.482 ± 0.041 | 1.0.E-03 |

**Supplementary Table S12. Enzyme activity validation of TetA mutants predicted by epiSCANEER.** Specific growth rate fold-change indicates the mean relative specific growth rate compared to wild-type. All data represent biological replicates (*n* = 3) with standard deviations. *p*-values indicate the statistical significance of titer fold-change measured by two-tailed Student's *t*-test, where "ns" denotes not significant (*P* > 0.05).

**Reference**

1. Lim,H.G., Kwak,D.H., Park,S., Woo,S., Yang,J.S., Kang,C.W., Kim,B., Noh,M.H., Seo,S.W. and Jung,G.Y. (2019) Vibrio sp. dhg as a platform for the biorefinery of brown macroalgae. *Nat. Commun.*, **10**.

2. Seok,J.Y., Yang,J., Choi,S.J., Lim,H.G., Choi,U.J., Kim,K.-J., Park,S., Yoo,T.H. and Jung,G.Y. (2018) Directed evolution of the 3-hydroxypropionic acid production pathway by engineering aldehyde dehydrogenase using a synthetic selection device. *Metab. Eng.*, **47**, 113–120.

3. Woo,S., Lim,H.G., Han,Y.H., Park,S., Noh,M.H., Baek,D., Moon,J.H., Seo,S.W. and Jung,G.Y. (2022) A Vibrio-based microbial platform for accelerated lignocellulosic sugar conversion. *Biotechnol. biofuels bioprod.*, **15**, 58.
